# Phenotype of Peripheral NK Cells in Latent, Active, and Meningeal Tuberculosis

**DOI:** 10.1155/2021/5517856

**Published:** 2021-04-27

**Authors:** José Alberto Choreño-Parra, Luis Armando Jiménez-Álvarez, Ellis Daniela Maldonado-Díaz, Graciela Cárdenas, Luis Alejandro Fernández-Lopez, José Luis Soto-Hernandez, Marcela Muñoz-Torrico, Gustavo Ramírez-Martínez, Alfredo Cruz-Lagunas, Armando Vega-López, María Lilia Domínguez-López, Carlos Sánchez-Garibay, Parménides Guadarrama-Ortíz, Silvia Giono, Luis Antonio Jiménez-Zamudio, Shabaana A. Khader, Ethel A. García-Latorre, Citlaltepetl Salinas-Lara, Joaquín Zúñiga

**Affiliations:** ^1^Instituto Politécnico Nacional, Escuela Nacional de Ciencias Biológicas, Laboratorio de Inmunoquímica I, Mexico City, Mexico; ^2^Laboratory of Immunobiology and Genetics, Instituto Nacional de Enfermedades Respiratorias “Ismael Cosío Villegas”, Mexico City, Mexico; ^3^Neuroinfectology Department, Instituto Nacional de Neurología y Neurocirugía “Manuel Velasco Suárez”, Mexico City, Mexico; ^4^Tuberculosis Clinic, Instituto Nacional de Enfermedades Respiratorias “Ismael Cosío Villegas”, Mexico City, Mexico; ^5^Instituto Politécnico Nacional, Escuela Nacional de Ciencias Biológicas, Laboratorio de Toxicología Ambiental, Mexico City, Mexico; ^6^Department of Neuropathology, Instituto Nacional de Neurología y Neurocirugía “Manuel Velasco Suárez”, Mexico City, Mexico; ^7^Centro Especializado en Neurocirugía y Neurociencias México (CENNM), Mexico City, Mexico; ^8^Instituto Politécnico Nacional, Escuela Nacional de Ciencias Biológicas, Departamento de Microbiología, Mexico City, Mexico; ^9^Department of Molecular Microbiology, Washington University School of Medicine in St. Louis, St. Louis, MO, USA; ^10^Escuela de Medicina y Ciencias de la Salud, Tecnologico de Monterrey, Mexico City, Mexico

## Abstract

The mechanisms underlying the immunopathology of tuberculous meningitis (TBM), the most severe clinical form of extrapulmonary tuberculosis (TB), are not understood. It is currently believed that the spread of *Mycobacterium tuberculosis* (Mtb) from the lung is an early event that occurs before the establishment of adaptive immunity. Hence, several innate immune mechanisms may participate in the containment of Mtb infection and prevent extrapulmonary disease manifestations. Natural killer (NK) cells participate in defensive processes that distinguish latent TB infection (LTBI) from active pulmonary TB (PTB). However, their role in TBM is unknown. Here, we performed a cross-sectional analysis of circulating NK cellCID="C008" value="s" phenotype in a prospective cohort of TBM patients (*n* = 10) using flow cytometry. Also, we addressed the responses of memory-like NK cell subpopulations to the contact with Mtb antigens *in vitro*. Finally, we determined plasma levels of soluble NKG2D receptor ligands in our cohort of TBM patients by enzyme-linked immunosorbent assay (ELISA). Our comparative groups consisted of individuals with LTBI (*n* = 11) and PTB (*n* = 27) patients. We found that NK cells from TBM patients showed lower absolute frequencies, higher CD69 expression, and poor expansion of the CD45RO^+^ memory-like subpopulation upon Mtb exposure *in vitro* compared to LTBI individuals. In addition, a reduction in the frequency of CD56^bright^CD16^−^ NK cells characterized TBM patients but not LTBI or PTB subjects. Our study expands on earlier reports about the role of NK cells in TBM showing a reduced frequency of cytokine-producing cells compared to LTBI and PTB.

## 1. Introduction

*Mycobacterium tuberculosis* (Mtb), the causative agent of tuberculosis (TB), remains the leading cause of death associated with a single pathogen [1]. Approximately a quarter of the world population has latent TB infection (LTBI) [2], and 10% of the infected individuals are at risk of developing active pulmonary TB (PTB) [1]. The limited protective effectiveness of the bacillus Calmette-Guerin (BCG) TB vaccine contributes to this global crisis. Moreover, the broad clinical spectrum of TB delays the diagnosis and initiation of antibiotic therapy, thus impeding an adequate control of Mtb transmission. In this regard, different clinical scenarios can result from human-Mtb interactions. As mentioned above, 90% of infected humans with LTBI develop adaptive immune responses that control but do not eliminate Mtb, remaining asymptomatic. Another group of Mtb-infected individuals cannot establish or maintain protective immune mechanisms, thus progressing to active PTB. From these, most individuals manifest clinical data of Mtb infection limited to the lung, whereas in a small group of TB patients, the bacillus spreads to extrapulmonary organs [3].

Tuberculous meningitis (TBM) is the most severe form of extrapulmonary TB due to its high morbidity and mortality rates [4]. Unfortunately, the factors controlling the Mtb dissemination into the central nervous system (CNS) and the immunopathology of TBM are not completely defined [5]. The current understanding of the immune determinants of the clinical outcome of TB is based on the study of T cell adaptive immune responses. This approach has revealed novel correlates of protection which do not always provide sterilizing immunity in animal models and have shown low prognostic value to predict disease progression in LTBI individuals [6, 7]. More recently, targeting diverse components of the innate immune system has emerged as an attractive approach for TB vaccine development [8–11]. This strategy is based on novel discoveries about the importance of specific myeloid cell subtypes and innate lymphoid cell (ILC) subsets for protective immunity against Mtb.

NK cells are innate lymphocytes that exert cytotoxic and cytokine-production activities and can mediate recall responses against previously recognized stimuli, resembling memory lymphocytes [12]. Therefore, these cells are crucial for immune responses against several pathogens, including Mtb [9, 13]. NK cells infiltrate the lungs of PTB patients and can respond to contact with the bacillus *in vitro* [14–18]. In animal TB models, these cells can compensate for the absence of adaptive lymphocytes, mediating early effector activities that control the pulmonary infection with Mtb [19]. Several phenotypical and functional deficiencies are displayed by peripheral NK cells from PTB patients compared to LTBI individuals, supporting a role for NK cells in defenses against pulmonary Mtb [15, 20–24]. Strikingly, NK cell subsets with adaptive properties expand in mice, primates, and humans with TB, making them potential targets for vaccines [25–28]. However, the phenotype and function of NK cells in TB patients that develop extrapulmonary disease manifestations, including TBM, has not been extensively addressed. This is important, since the dissemination of Mtb is an early event during which NK cells and other innate immune cells may participate [29].

Here, we characterized the immunophenotype of circulating NK cells in patients with TBM and compared it with LTBI and PTB subjects. Our results provide novel insights into the role of NK cells in immunity against Mtb.

## 2. Materials and Methods

### 2.1. Human Participants

We conducted a prospective study in adult patients with acute TBM that attended and were admitted to the Neuroinfectology Department of the Instituto Nacional de Neurología y Neurocirugía Manuel Velasco Suarez (INNyN), in Mexico City, from January of 2017 to December of 2018. Only those individuals that met the clinical criteria for probable or definitive TBM, according to the case definition established in Cape Town, South Africa, in 2009 [30], were eligible.

Peripheral blood samples were obtained from enrolled participants on admission. Our comparative cohorts included LTBI and PTB patients recruited at the TB clinic of the Instituto Nacional de Enfermedades Respiratorias Ismael Cosío Villegas (INER), in Mexico City. The LTBI group included healthy close contacts of PTB patients with positive results in the QuantiFERON®-TB Gold Plus test (QIAGEN, Hilden, Germany). The PTB group included patients with laboratory-confirmed TB diagnosis by positive results in sputum smear microscopy, sputum/bronchoalveolar lavage (BAL) culture, and GeneXpert MTB/RIF test (Cepheid, CA, USA). A group of age- and sex-matched healthy volunteer donors was recruited and considered as controls (HC).

Solid-organ transplant recipients and patients with human immunodeficiency virus (HIV) infection, receiving immunosuppressive treatment, diagnosed with cancer, diabetes, or autoimmune diseases, were excluded from the study. Clinical and demographic data from participants were obtained by direct clinical interview, physical examination, and review of their medical records.

### 2.2. Sample Processing

Peripheral blood mononuclear cells (PBMCs) were isolated by centrifugation gradient using Ficoll-Paque™ PLUS (GE Healthcare, Life Sciences, PA, USA) as described before. Plasma aliquots for protein determinations were stored at -80°C until use.

### 2.3. *In Vitro* Assays

Freshly isolated PBMCs from HC, LTBI, PTB, and TBM individuals were exposed to Mtb antigens *in vitro* as previously described [15]. Briefly, cells were plated at a density of 2.5 × 10^6^ cells per mL in complete Roswell Park Memorial Institute (RPMI-1640) medium supplemented with 2 mM L-glutamine and 10% fetal bovine serum (FBS) and cultured with 25 *μ*g/mL of a cell wall (CW) extract of Mtb H37Rv at 37°C, 5% CO_2_, for 48 hours. The H37Rv CW preparation was gently provided by Dr. Shabaana A. Khader, from the Department of Molecular Microbiology, Washington University School of Medicine in St Louis, MO, USA.

### 2.4. Flow Cytometry

Freshly isolated or Mtb H37Rv CW-stimulated PBMCs were stained with appropriate dilutions of the following specific fluorochrome-labeled antibodies: BV510 anti-human CD3 (OKT3, BioLegend, USA), BV510 anti-human CD14 (M5-E2, BioLegend, USA), PerCP anti-human CD56 (HCD56, BioLegend, USA), APC/Cy7 anti-human CD16 (3G8, BioLegend, USA), APC anti-human NKG2D (1D11, BioLegend, USA), PE anti-human NKp46 (9E2, BioLegend, USA), BV421 anti-human CD69, (FN50, BioLegend, USA), FITC anti-human CD45RO (UCHL1, BioLegend, USA), and AlexaFluor700™ anti-human CD27 (O323, 302814, USA). After staining, samples were washed with Cell Staining Buffer (BioLegend, 420201, USA), resuspended in 4% paraformaldehyde, and acquired in a BD FACS™ Aria II cytometer (BD Biosciences, USA) using FACSDiva software. Tubes with microbeads (Anti-Mouse Ig, *κ*/Negative Control Compensation Particles Set, BD™ CompBead, BD Biosciences, USA) were stained with single fluorochrome-labeled antibodies and served to set a compensation matrix. Cells were gated based on their forward/side scatter characteristics and a fluorescence minus one (FMO) control for each specific marker. Human NK cells were defined as CD3^−^CD14^−^CD56^+^. We acquired at least 1 × 10^4^ CD3^−^CD14^−^CD56^+^ NK cells from each sample. The compensation set up and calculation of the frequency of specific cell subsets were made using FlowJo (FlowJo, LLC, Ashland, OR, USA).

### 2.5. Plasma Protein Quantifications

Plasma levels of MHC class I polypeptide-related sequence A (MIC-A), MHC class I polypeptide-related sequence B (MIC-B), and UL16 binding protein 1 (ULBP-1) were determined by enzyme-linked immunosorbent assay (ELISA) using commercial kits (MBS175982, MBS177192, and MBS3800229, MyBioSource, USA), and following the manufacturer's instructions.

### 2.6. Study Approval

The current study was reviewed and approved by the Institutional Review Board of the INER (project number B04-15) and the Ethics Committee of the INNyN (project number 160/16) in Mexico City. All patients or their legal guardians provided written consent to participate in the study. Blood samples were processed and stored according to the Mexican Constitution law NOM-012-SSA3-2012, which establishes criteria for executing clinical research projects in humans.

### 2.7. Statistical Analysis

Descriptive statistics were used to characterize the study population clinically. Specific tests are mentioned in figure and table legends. Statistical analyses were performed using GraphPad Prism 8 (La Jolla, CA, USA). Two-tailed *p* values ≤ 0.05 were considered as significant: ^∗^*p* ≤ 0.05, ^∗∗^*p* ≤ 0.01, ^∗∗∗^*p* ≤ 0.001, and ^∗∗∗∗^*p* ≤ 0.0001.

## 3. Results

### 3.1. Participant Characteristics

TBM is an infrequent but severe complication of extrapulmonary TB [4]. As such, we were able to recruit only ten patients with TBM over two years for the present study. From these patients, six were females and four males, with a median age of 35 years. Our comparative cohorts consisted of 27 patients with active PTB and 11 individuals with LTBI. Their main clinical features are summarized in Table 1. Thirty-seven percent of PTB participants were infected with multi-drug resistant (MDR) Mtb strains. Meanwhile, four patients in the TBM group met the criteria for a definitive disease, as the infection was confirmed by positive culture of cerebrospinal fluid (CSF). The remaining six patients were categorized as probable TBM, according to their clinical, radiological, and laboratory test characteristics [30], which are further described in Table 2. Enrolled patients with probable and definitive TBM presented meningeal signs (70%), fever (50%), motor deficit (50%), sensitive deficit (30%), and cranial nerve palsies (30%) as their main clinical manifestations. Also, TBM patients typically showed lymphocytic pleocytosis, low glucose levels, elevated proteins, and increased adenosine deaminase (ADA) in the CSF analysis, as well as vasculitis, hydrocephalus, and basal meningeal enhancement in the brain magnetic resonance imaging (MRI; see Table 2). Two TBM patients died due to severe neurological manifestations. Interestingly, most recruited participants with meningitis denied a history of PTB, and chest X-ray images obtained at hospital admission showed no lung involvement in six TBM patients. This supports a possible neurotropism of some Mtb strains, as suggested before [31].

### 3.2. Peripheral NK Cell Subpopulations in TBM Patients

**Figure 1:**
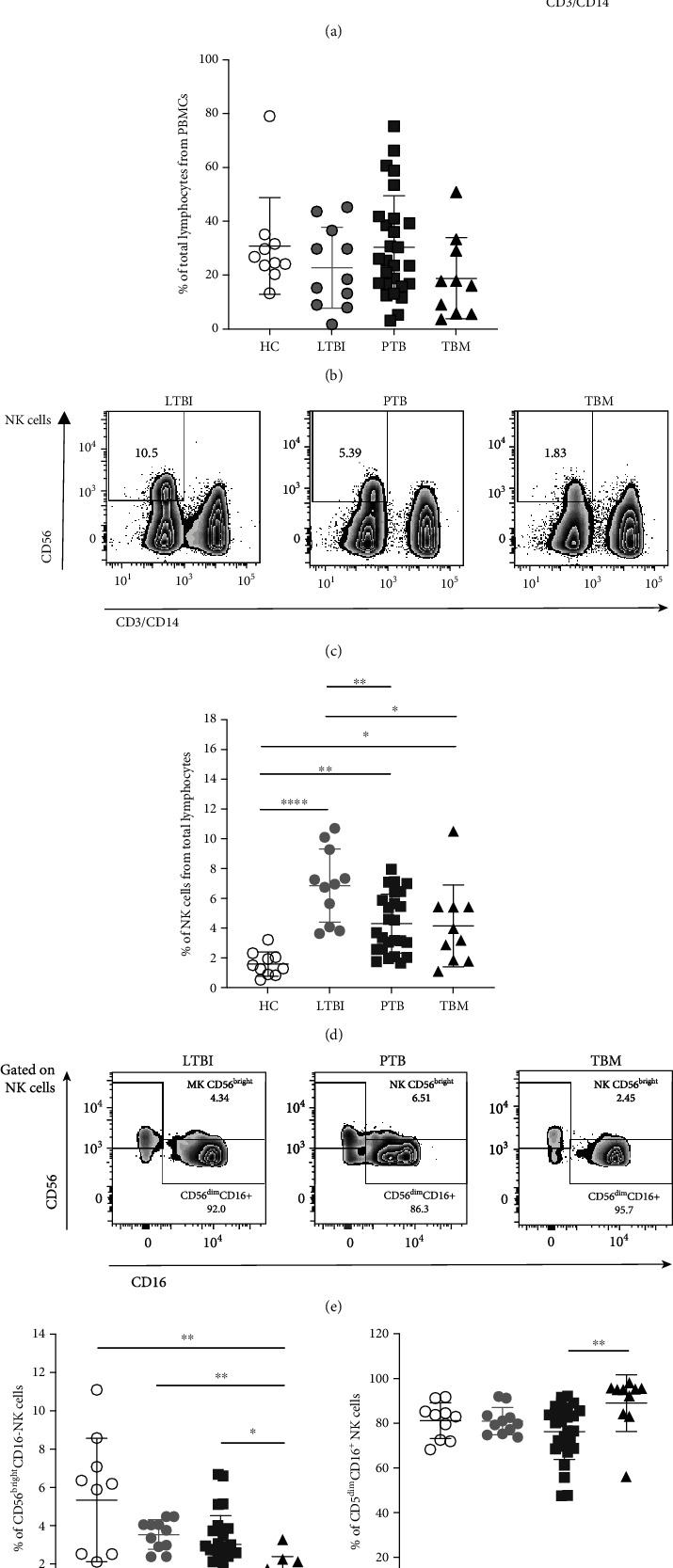
Major NK cell subpopulations in patients with TBM. (a) Flow cytometry gating strategy for the analysis of circulating NK cells in peripheral blood mononuclear cell (PBMC) samples from healthy controls (HC, *n* = 10), individuals with latent TB infection (LTBI, *n* = 11), patients with active pulmonary TB (PTB, *n* = 27), and patients with tuberculous meningitis (TBM, *n* = 10). (b) Percentage of lymphocytes from total PBMCs. (c, d) Percentage of NK cells from total lymphocytes. (e) Analysis of major NK cell subpopulations in the blood. (f) Percentage of CD56^bright^CD16^−^ NK cells. (g) Percentage of CD56^dim^CD16^+^ NK cells. Differences between groups were analyzed using the Kruskal-Wallis test and the post hoc Dunn's test for multiple comparisons. The data shown represent the mean (±SE) values. ^∗^*p* ≤ 0.05, ^∗∗^*p* ≤ 0.01, and ^∗∗∗^*p* ≤ 0.001.

Previous investigations addressing the role of NK cells in human TB have revealed phenotypical deficiencies in PTB patients compared to LTBI individuals [15, 20–24], suggesting the protective participation of NK cells during pulmonary Mtb infection. However, little evidence exists about the phenotype of these cells in patients with extrapulmonary manifestations of the disease. Here, we focused part of our study on determining the relative frequency of some of the main NK cell subpopulations in humans with TBM and made a comparison with LTBI and PTB subjects. For this purpose, we use flow cytometry in PBMC samples obtained from all study participant groups.  Figure 1(a) shows the gating strategy used for enumerating NK cells.

Our analyses showed no differences in the percentage of total lymphocytes in PBMCs between groups (Figure 1(b)). Strikingly, NK cells were significantly less abundant in TBM patients (3.57%, 1.83%-5.42%, interquartile range [IQR]) compared to LTBI individuals (6.95%, 4.07%-9.28%, IQR, *p* = 0.0232). Similarly, PTB patients also showed lower percentages of total NK cells (3.7%, 2.57%-6.13%, IQR) than LTBI subjects (*p* = 0.0074; Figures 1(c) and 1(d)). These findings coincide with previous reports of diminished amounts of total NK cells as a hallmark of active pulmonary Mtb infection in humans [15, 20–24]. Hence, our results demonstrate that circulating NK cells are also depleted from the circulation in patients with extrapulmonary manifestations of TB, like TBM.

One of the principal alterations observed in PTB patients is the reduced frequency of CD56^bright^CD16^−^ NK cells in the peripheral blood [23]. This subpopulation is characterized by a lower maturation state and a higher capacity to produce cytokines upon stimulation [32]. We also analyzed the frequency of these cells in our cohorts of TB patients. As expected, the percentages of CD56^bright^CD16^−^ NK cells were slightly lower in PTB (2.75%, 2.11%-3.73%, IQR) than those in LTBI subjects (3.76%, 2.9%-4.06%, IQR), although the difference did not reach statistical significance (Figures 1(e) and 1(f)). Remarkably, CD56^bright^CD16^−^ NK cells were diminished in TBM patients (1.32%, 0.69%-2.15%, IQR) compared to LTBI (*p* = 0.0025) and PTB patients (*p* = 0.0331). Conversely, the TBM group differed from the rest of the participants regarding their higher percentages of NK cells belonging to the CD56^dim^CD16^+^ subpopulation (Figure 1(g)). These cells are mature and possess an intrinsic cytotoxic function [32]. Of note, the percentage of CD56^dim^CD16^+^ correlated with the body mass index (BMI) of TBM patients (Figure S1).

**Figure 2:**
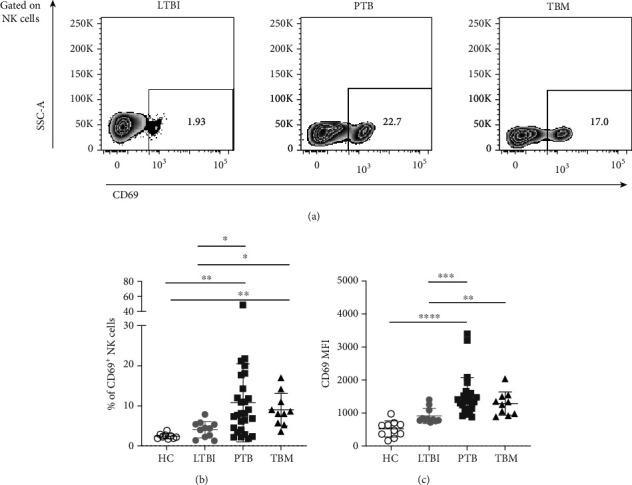
Expression of CD69 in peripheral NK cells from patients with TB. (a) The expression of CD69 in peripheral blood NK cells from healthy controls (HC, *n* = 10), individuals with latent TB infection (LTBI, *n* = 11), patients with active pulmonary TB (PTB, *n* = 27), and patients with tuberculous meningitis (TBM, *n* = 10) was assessed by flow cytometry. (b) Percentage of CD69^+^ NK cells. (c) Mean fluorescence intensity (MFI) values for CD69 in NK cells. Differences between groups were analyzed using the Kruskal-Wallis test and post hoc Dunn's test for multiple comparisons. The data shown represent the mean (±SE) values. ^∗^*p* ≤ 0.05 and ^∗∗∗^*p* ≤ 0.001.

Together, our results show that CD56^bright^CD16^−^ NK cells are reduced in the circulation of TBM patients, and such a reduction is more significant than in PTB subjects (1.32%, 0.69%-2.15%, IQR vs. 2.75%, 2.11%-3.73%, IQR, *p* = 0.0331). These findings may also indicate an active mobilization of NK cells from the circulation to the sites of infection in TBM patients. Therefore, we evaluated the expression of the activation and tissue-homing marker CD69 in peripheral NK cells [33]. Notably, we found a significantly higher percentage of CD69^+^ NK cells in TBM patients (8.34%, 5.64%-11.8%, IQR) compared to HC (2.24%, 1.89%-2.77%, IQR, *p* = 0.0025) and a slight difference with respect to LTBI individuals (4.38%, 2.23%-5.4%, IQR, *p* = 0.0737; Figures 2(a) and 2(b)). Furthermore, NK cells from TBM patients showed a higher CD69 mean fluorescence intensity (MFI) than LTBI subjects (*p* < 0.05; Figure 2(c)). Similar observations were made in PTB patients, whereas there were no differences in the MFI and the percentage of CD69^+^ NK cells between TBM and PTB groups.

We also compared the phenotype of circulating NK cells between probable and definite TBM patients, since both groups might differ in Mtb burden and thus detectability of the infection. We found that patients with definite disease showed reduced frequencies of CD56^dim^CD16^+^ NK cells (Figure S1). Meanwhile, no differences in total, CD56^bright^CD16^−^, and CD69^+^ NK cells were observed between TBM patients. Also, no correlations between NK cell subsets and prognostic variables such as the Glasgow Coma Scale (GCS) and British Medical Research Council stage at admission were observed (Figure S1).

### 3.3. Expression of Surface-Activating Receptors in Peripheral NK Cells

A variety of activating and inhibitory receptors govern the functions of NK cells [34]. Some of these receptors allow NK cells to recognize pathogen-associated molecular patterns (PAMPs) and exert effector functions against infective agents [17, 35, 36]. As such, the deficient expression of activating receptors may limit the capacity of NK cells to respond during infections. Thus, we also evaluated the expression of activating NK cell receptors in our patients to determine if the lower control and higher severity of infection in TBM patients were related to possible phenotypical alterations of NK cells.

**Figure 3:**
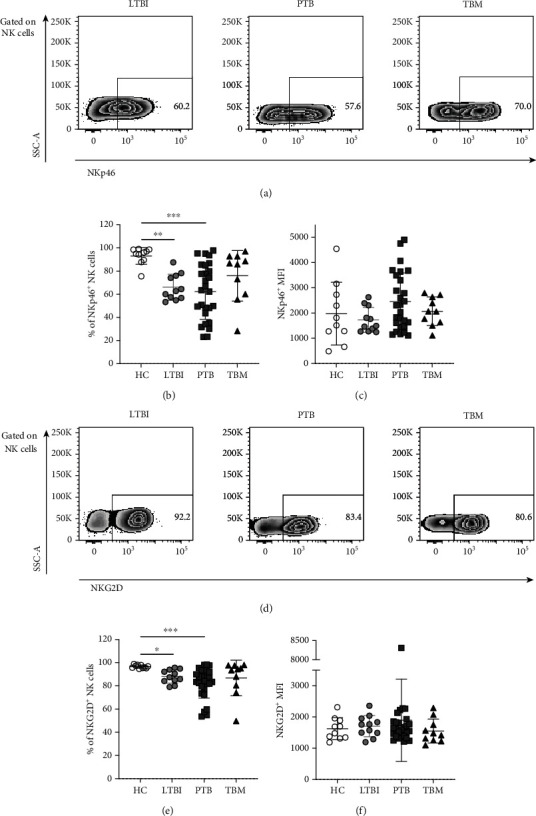
Expression of activating receptors in peripheral blood NK cells from patient with TBM. (a) Analysis of the expression of NKp46 in peripheral blood NK cells from healthy controls (HC, *n* = 10), individuals with latent TB infection (LTBI, *n* = 11), patients with active pulmonary TB (PTB, *n* = 27), and patients with tuberculous meningitis (TBM, *n* = 10). (b) Percentage of NKp46^+^ NK. (c) Mean fluorescence intensity (MFI) values for NKp46 in NK cells. (d) Analysis of the expression of NKG2D in peripheral blood NK cells. (e) Percentage of NKG2D^+^ NK cells. (f) Mean fluorescence intensity (MFI) values for NKG2D in NK cells. Differences between groups were analyzed using the Kruskal-Wallis test and post hoc Dunn's test for multiple comparisons. The data shown represent the mean (±SE) values.

First, we analyzed the expression of the natural killer cell p46-related protein (NKp46) receptor, which is a member of the natural cytotoxicity receptor (NCR) family. NKp46 mediates the recognition and lysis of Mtb-infected human monocytes after binding to vimentin [37]. Thus, a deficiency in the expression of NKp46 may render NK cells incapable of eliminating intracellular reservoirs of Mtb infection. Interestingly, we found a reduction of NKp46^+^ NK cells in the LTBI and PTB groups compared to HC, which coincides with a previous report describing significant downregulation of NKp46 in LTBI individuals [38]. However, we did not observe any difference in the percentage of NKp46^+^ NK cells relative expression of this marker between TBM, PTB, and LTBI patients (Figures 3(a)–3(c)). We also compared the expression of the natural killer group 2 member D (NKG2D) C-type lectin-like receptor between participant groups. This molecule mediates the recognition and elimination of Mtb-infected monocytes upon attachment to the ULBP-1 ligand on their surface [18]. As for NKp46, NKG2D^+^ NK cells were more abundant in HC, but we did not find any difference in the percentage of NKG2D^+^ NK cells and the relative expression of this molecule between TBM patients and the other TB groups (Figures 3(d)–3(f)).

These findings suggest that differences in TB disease susceptibility are not related with deficiencies in the expression of NKp46 and NKG2D. Hence, the participation of NK cells during TBM, if any, is not dependent on NKp46- and NKG2D-mediated cytotoxicity against infected phagocytes *in vivo*. This is in sharp contrast with evidence of the Mtb-induced upregulation of ligands for activating NK cell receptors in infected cells *in vitro*. For instance, as mentioned above, human monocytes infected with Mtb increase the expression of the NKG2D receptor ligand ULBP-1 [18]. Similarly, Mtb-infected dendritic cells (DCs) also upregulate the molecule MIC-A in their surface [39], which is also recognized by NKG2D. Interestingly, a lower frequency of both NKp46^+^ and NKG2D^+^ NK cells was found in patients with definite but not probable TBM (Figure S1). This finding might imply that deficiencies in the expression of activating receptors are related to higher Mtb burden among TBM patients despite not being associated with higher risk of disseminated disease in the overall TB population.

**Figure 4:**
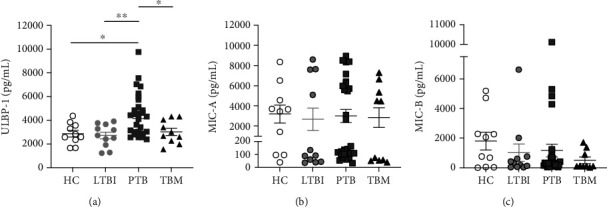
Soluble ligands of the NKG2D receptor in the serum of patients with TB. Serum samples from healthy controls (HC, *n* = 10), individuals with latent TB infection (LTBI, *n* = 11), patients with active pulmonary TB (PTB, *n* = 27), and patients with tuberculous meningitis (TBM, *n* = 10) were used for determinations of the levels of soluble NKG2D ligands by ELISA. (a) Serum levels of ULBP-1. (b) Serum levels of MIC-A. (c) Serum levels of MIC-B. Differences between groups were analyzed using the Kruskal-Wallis test and post hoc Dunn's test for multiple comparisons. The data shown represent the mean (±SE) values. ^∗^*p* ≤ 0.05 and ^∗∗^*p* ≤ 0.01.

Infected and malignant cells can escape from the activity of NKG2D by shedding these ligands [40], which then act as decoy molecules that inactivate the cytotoxic capacity of NK cells. These soluble products may become detectable in the serum. To address whether this mechanism of immune evasion is employed by Mtb and operates during TBM, we measured the serum levels of three different soluble NKG2D receptor ligands: ULBP-1, MIC-A, and MIC-B. Of note, we found high serum levels of ULBP-1 only among PTB patients (4380 pg/mL, 3015 pg/mL–5538 pg/mL, IQR), but not in HC (2715 pg/mL, 2182 pg/mL–3640 pg/mL, IQR, *p* = 0.0178), LTBI (2729 pg/mL, 1929 pg/mL–3666 pg/mL, IQR, *p* = 0.0055), and TBM individuals (2847 pg/mL, 2198 pg/mL–4120 pg/mL, IQR, *p* = 0.0364; Figure 4(a)). In contrast, there were no differences in the levels of MIC-A and MIC-B between all participant groups (Figures 4(b) and 4(c)). These observations indicate that the evasion of NK cell cytotoxicity through the shedding of ULPB-1 from infected phagocytes may be an important mechanism in the pathogenesis of PTB but not TBM. However, we cannot rule out the participation of this phenomenon inside the Mtb-infected brain during TBM, as we did not explore the CSF levels of these ligands. Collectively, our results suggest that NK cell responses are differentially regulated during TBM, PTB, and LTBI.

### 3.4. Memory-Like NK Cells in Humans with TBM

A striking functional property of NK cells is their ability to mediate secondary responses against antigenic and nonantigenic stimuli, resembling memory of adaptive lymphocytes. This mechanism provides protection against viruses in mice and might participate in immunity to human infections [12]. Indeed, several subpopulations of memory-like NK cells might get involved in the mechanisms of defense during TB [8, 9]. For instance, BCG-vaccinated mice display an IL-21 dependent expansion of CD27^+^ NK cells that mediate protective memory-like responses against Mtb [27]. These CD27^+^ NK cells are also expanded in LTBI patients but not healthy individuals and proliferate upon *in vitro* exposure to Mtb.

**Figure 5:**
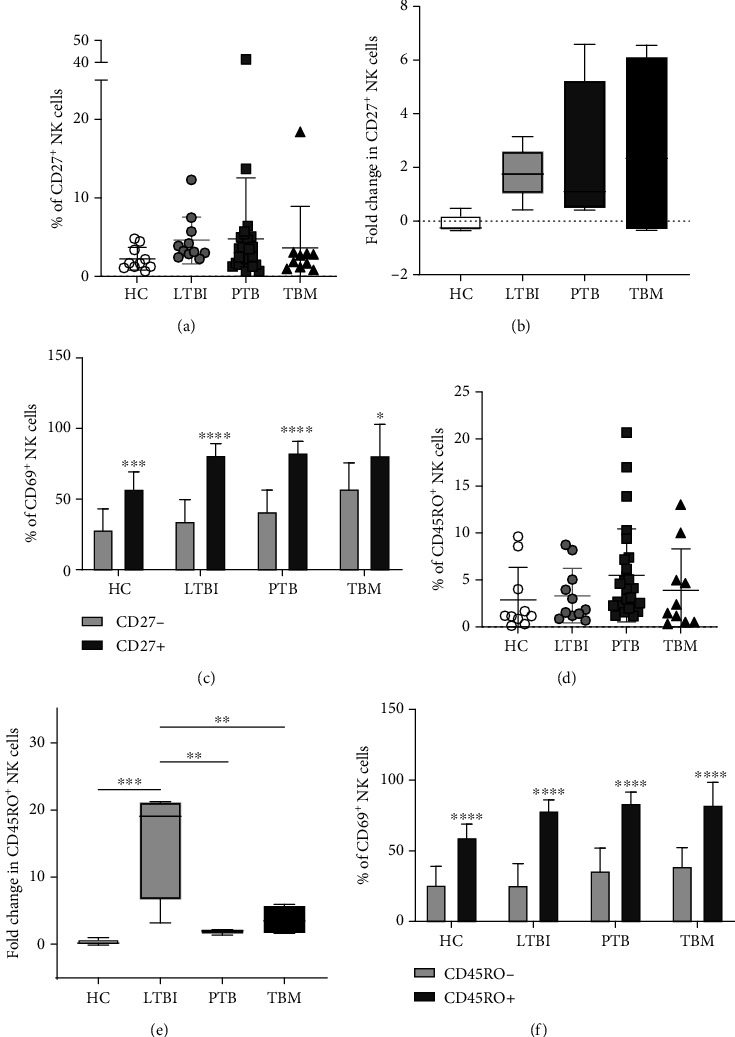
Memory-like NK cells in patients with TBM. (a) The percentage of CD27^+^ NK cells in peripheral blood mononuclear cell (PBMC) samples from healthy controls (HC, *n* = 10), individuals with latent TB infection (LTBI, *n* = 11), patients with active pulmonary TB (PTB, *n* = 27), and patients with tuberculous meningitis (TBM, *n* = 10) was determined by flow cytometry. (b) PMBCs from HC, LTBI, PTB, and TBM patients were cultured with a cell wall (CW) extract of Mtb H37Rv for 48 h (*n* = 5 per group). After the stimulation, the percentage and fold increase of CD27^+^ NK cells were determined. (c) The proportion of CD69^+^ cells was compared between CD27^+^ and CD27^−^ NK cells at each group. (d) Percentage of peripheral blood CD45RO^+^ NK cells. (e) After the stimulation, the percentage and fold increase of CD45RO^+^ NK cells were determined. (f) The proportion of CD69^+^ cells was compared between CD45RO^+^ and CD45RO^−^ NK cells at each group. Fold increases were calculated as follows: the percentage of a specific cell subpopulation after culture of PBMCs with Mtb antigens was divided by the percentage of the same cell subset before such stimulation. Differences between groups were analyzed using the Kruskal-Wallis test and post hoc Dunn's test for multiple comparisons. Comparisons between cells from the same group were analyzed with the Student *t*-test and *p* values corrected for multiple comparisons using the Holm method. The data shown represent the mean (±SE) values. ^∗^*p* ≤ 0.05, ^∗∗^*p* ≤ 0.01, ^∗∗∗^*p* ≤ 0.001, and ^∗∗∗∗^*p* ≤ 0.0001.

To address whether CD27^+^ NK cells are relevant during TBM, we determined the relative frequency of these cells in the circulation of our study participants. However, we did not find differences in the percentage of circulating CD27^+^ NK cells between HC, LTBI, PTB, and TBM patients (Figure 5(a)). We also compared the *in vitro* responses of CD27^+^ NK cells against the contact with a CW extract of Mtb H37Rv. As previously reported [27], we found that cells from LTBI, but not healthy individuals, proliferate upon exposure to Mtb antigens (Figure 5(b)). CD27^+^ NK cells also expanded in PTB and TBM, although to a lower level than LTBI individuals. Interestingly, CD27^+^ NK cells expressed the activation and tissue-homing marker CD69 with higher frequency than CD27^−^ NK cells in all participant groups (Figure 5(c)). This finding suggests that CD27^+^ NK cells have an intrinsic higher capacity to respond to Mtb antigens, but their responsiveness is similar in PTB and TBM patients. Hence, these data indicate that CD27^+^ NK cells might not be relevant for protection against extrapulmonary manifestations of TB, such as TBM.

Another subgroup of adaptive NK cells that express the memory marker CD45RO has been isolated from the pleural fluid of individuals with tuberculous pleural effusion [25, 26]. These cells show increased cytotoxic and cytokine production capacity in response to IL-12 and BCG as compared to their CD45RO^−^ counterpart. As for CD27^+^ NK cells, we also compared the frequency of CD45RO^+^ NK cells in the blood of HC and subjects with LTBI, PTB, and TBM. This analysis showed no differences in the percentage of CD45RO^+^ NK cells between all participant groups (Figure 5(d)), although CD45RO^+^ NK cells were more abundant in the blood of patients with definite but not probable TBM (Figure S1). Remarkably, after an *in vitro* stimulation with Mtb H37Rv CW, an expansion of CD45RO^+^ NK cells was observed only among LTBI individuals but not patients with PTB and TBM (Figure 5(e)). Moreover, a higher percentage of CD45RO^+^ NK cells expressed CD69 than CD45RO^−^ NK cells in response to Mtb antigens in all participants (Figure 5(f)). Collectively, these data indicate that memory-like CD45RO^+^ NK cells respond to Mtb antigens and may play a protective role during TB. However, the responses of these cells do not impact the risk of progression of PTB to TBM.

## 4. Discussion

TB of the CNS encompasses a spectrum of manifestations that includes meningitis, parenchymal tuberculomas, tuberculous abscesses, and vasculitis. These entities are characterized by an intense inflammatory response that can cause severe nervous tissue damage [4, 5]. TBM is the most devastating form of extrapulmonary TB due to its high mortality and neurological sequela. Despite this, the immunopathogenesis of TBM is not completely understood so far. Much of what is currently known relies on descriptions made by Rich and McCordock almost a century ago [41]. These researchers proposed that Mtb could reach the CNS a long time before infected individuals manifest symptoms remaining silent within the brain. Nonetheless, the route and mechanisms by which Mtb invades the human brain remained unclear for many years until recent advances were achieved using *in vitro* assays and animal models [42–44].

The CNS is separated from the systemic circulation by the blood-brain barrier (BBB) and the blood-cerebrospinal fluid barrier (BCSFB). These barriers limit the access of circulating substances and infectious agents to the nervous system [45]. Nevertheless, certain neuroinfectious pathogens have virulence factors that allow them to adhere to the endothelium and cross the BBB [46, 47]. Indeed, some clinical strains of Mtb isolated from humans with TBM can cause CNS infection after intratracheal inoculation to mice [31]. Also, *in vitro* assays have demonstrated that Mtb can cross the BBB via transcytosis [42]. The pathogen might also enter the brain as free mycobacteria or inside an infected monocyte [43, 44], and specific cytokines induced during the infection might make BBB more permeable to Mtb [46, 47].

Along with these mechanisms, the dissemination of TB to the CNS also depends on lung defenses' ability to control the initial infection. Indeed, some investigations suggest that the spread of Mtb to extrapulmonary organs is a silent and very early event that precedes the initiation of antigen-specific adaptive immune responses in the lung [29]. Hence, the function of a plethora of innate defense mechanisms might determine the disease's course and progression. Among these innate components of immunity, NK cells play a relevant role during PTB. Their participation and specific effector functions that improve the control of Mtb have been extensively revised elsewhere [8, 9, 13]. Despite this, little literature exists on the role of NK cells in the brain inflammatory response associated with TBM. This is in part related to the lack of animal models that mimic the spread of pulmonary TB to the CNS since those that exist use the intracranial or intravenous route to inoculate the pathogen into the brain [48]. Furthermore, due to the low frequency and complicated differential diagnosis of the disease, samples from humans with TBM are scarcely available to be analyzed at the early stages of infection.

NK cells might play a role in the pathogenesis of different viral and bacterial infections of the CNS. In some cases, such as brain infection with herpes viruses, the activity of NK cells is protective [49, 50]. As such, children with functional and genetic deficiencies in NK cells are susceptible to herpetic encephalitis [49], while mice completely deployed of NK cells and T cells show more severe encephalitis than animals only deficient of T cells after inoculation with herpes simplex virus type 1 (HSV-1) [50]. The cytotoxic function of NK cells protects against a neurovirulent strain of the simian immunodeficiency virus (SIV) in macaques [51] and cerebral malaria in humans [52]. Also, the cytokine production activity of NK cells is crucial against *Listeria monocytogenes* neuroinvasion in mice [53], but pathogenic during *Streptococcus pneumoniae* meningitis [54]. Despite these data, studies addressing the relevance of NK cells during neuroinfections remain scarce.

In this context, our study is among the first ones that evaluated a possible role for NK cells during TBM in humans, providing novel evidence for the field. Our findings demonstrate that TBM and PTB patients showed similar phenotypical deficiencies in NK cells compared to LTBI individuals. Nonetheless, TB patients with CNS infection differ from those without neuroinvasion by specific alterations in circulating NK cells' phenotype. The most remarkable abnormality found only among TBM, but not in LTBI and PTB patients, was the lower amounts of total and cytokine-producing CD56^bright^ NK cells in the blood. As aforementioned, NK cells' cytokine production is pathogenic for some CNS bacterial infections [54]. Hence, the lack of total and CD56^bright^ NK cells in TBM patients may indicate, on the one hand, that these cells migrated to the CNS. Once inside the brain, these cells might produce proinflammatory cytokines that contribute to TBM patients' brain injury and clinical manifestations. On the other hand, the reduced number of CD56^bright^ NK cells in the blood could be a deficiency that contributes to the lack of control of Mtb infection in the lung, thus promoting its dissemination.

Conversely, TBM patients displayed a higher amount of cytotoxic CD56^dim^CD16^+^ NK cells in the circulation. Cytotoxicity mediated by NK cells is protective for some neurological complications of infections [51, 52], and increased numbers of cytotoxic NK cells in the lung are a hallmark that defines latency over active disease in macaques infected with Mtb [28]. Thus, the increased abundance of CD56^dim^CD16^+^ NK cells in TBM patients' blood might reflect an active mobilization of these cells to the CNS as an attempt to control the local infection. In fact, CD56^dim^CD16^+^ NK cells are reduced in the blood of human immunodeficiency virus- (HIV-) positive but not HIV-negative TBM patients, further supporting a possible protective role [55]. Alternatively, protective cytotoxic NK cells could get stuck in the blood, so they cannot move to local infection sites and contribute to eliminating the pathogen. In this regard, van Laarhoven and colleagues recently found that total NK cells are depleted from the blood but enriched in the CSF of a cohort of TBM patients [56], providing evidence that explains and coincides with some of our findings.

Interestingly, as reported in the mentioned study [56], we also found that NK cells from TBM patients highly express the tissue-homing marker CD69 to a similar level than NK cells from PTB subjects. In contrast, LTBI individuals showed a lower expression of CD69 in peripheral NK cells. These findings reveal that NK cells get activated and mobilize to tissues only during active but not latent Mtb infection. Hence, the expression of CD69 in different cells, not only NK cells, could be a readout that differentiates LTBI from active pulmonary and disseminated TB disease. Notably, although the expression of CD69 did not differ between TBM and PTB patients, a striking characteristic of our cohort of TBM patients was the absence of clinical and radiological data of pulmonary involvement on hospital admission. Hence, the expression of CD69 in NK cells during acute meningitis reflects an active mobilization to the CNS in our patients, as also reported by van Laarhoven and colleagues [56], who demonstrated that NK cells are among the main lymphoid cells enriched in the CSF of TBM patients. Notwithstanding, their study does not provide additional data about other phenotypical characteristics of NK cells during TBM.

Besides the main objectives of our work, we made two findings that may have important implications in the general understanding of anti-Mtb immunity. First, we found that, although memory-like CD45RO^+^ NK cells are not more abundant in the blood of LTBI as compared to patients with PTB and TBM, they are more responsive to the contact with Mtb antigens during latency. This fact has two possible explanations: (a) that CD45RO^+^ NK cells are more functional and participate in protective immunity in LTBI individuals, or (b) that NK cells with adaptive properties are depleted from the circulation of subjects with active TB as they are recruited to the sites of local Mtb infection. An analysis of the responsiveness of CD45RO^+^ NK cells from infected BAL and CSF samples would have clarified this point. However, as mentioned above, previous investigations have already demonstrated that CD45RO^+^ NK cells isolated from an active site of Mtb infection possess enhanced functional capacities [25, 26].

Secondly, we discover that higher levels of the soluble NKG2D ligand ULBP-1 are a readout that differentiates PTB from LTBI and TBM. This observation brings forward the unrecognized importance of the shredding of soluble NKGD2 ligands as an evasion mechanism of Mtb or as an immune defect associated with poor TB control in the lungs, but not in the CNS and during latency. This process operates in several cancers making tumors less susceptible to the antitumoral activity of NK cells. Hence, cells infected with Mtb might also be untargeted by NK cells keeping the intracellular reservoir of the infection intact, at least during the disease's initial stages. Moreover, our findings provide novel evidence in favor of the possible usage of serum ULBP-1 levels as a diagnostic biomarker to differentiate LTBI and PTB, which deserves further validation in future studies.

The main limitation of our work is the low number of TBM patients recruited. This is because, as we mentioned before, the incidence of this complication is low. Another flaw of this study is that we focused our analysis on peripheral blood NK cells, whereas previous investigations addressed their properties both in the circulation and in the local site of infection [56]. Thus, the protective or pathogenic nature of the role of NK cells during TBM is not completely apparent from our data. Also, additional functional assessment of NK cells would have provided complementary mechanistic information to support the observed differences. Finally, an important concern is that our experimental design does not allow the evaluation of a unique NK cell TBM signature because we did not include a non-TBM extrapulmonary TB control group.

However, as currently presented, our work represents an incremental advance in the field, since the phenotypical changes of peripheral NK cells observed in our cohort of TBM patients with respect to LTBI and PTB individuals reaffirm and complement previous findings suggesting an active role of these cells in immunity against pulmonary Mtb infection and prevention of its dissemination. Future studies should further evaluate the main activities of NK cells in brain tissue specimens or CSF samples from patients with TBM and animal models of the disease.

## Figures and Tables

**Table 1:** Participant characteristics.

Characteristic	LTBI (*n* = 11)	PTB (*n* = 27)	TBM (*n* = 10)	*p* values		
	A	B	C	A vs. B	A vs. C	B vs. C
Median age (range)	42 (19-80)	43 (18-67)	35 (21-52)	0.8458	0.4159	0.5831

Gender						
Female, *n* (%)	8 (72.72)	14 (51.85)	6 (60.0)	0.2960	0.6594	0.7246
Male, *n* (%)	3 (27.27)	13 (48.14)	4 (40.0)			

Weight, mean (SD)	70.91 (16.57)	54.41 (9.60)	71.39 (12.91)	0.0019	0.9816	0.0008

Height, mean (SD)	1.58 (0.08)	1.62 (0.08)	1.64 (0.09)	0.2572	0.2660	0.7416

BMI, mean (SD)	28.25 (5.71)	20.59 (3.59)	26.09 (3.88)	<0.0001	0.3369	0.0061

Drug resistance						
MDR, *n* (%)	ND	10 (37.03)	ND	—	—	—
Sensitive, *n* (%)	ND	14 (51.85)	ND	—	—	—
Undetermined, *n* (%)	ND	3 (11.11)	ND	—	—	—

MTB case category						
Definitive, *n* (%)	N/A	N/A	4 (40.0)	—	—	—
Probable, *n* (%)	N/A	N/A	6 (60.0)	—	—	—

Outcome						
Deceased, *n* (%)	0 (0.0)	0 (0.0)	2 (20.0)	—	—	—
Survived, *n* (%)	11 (100.0)	27 (100.0)	8 (80.0)	—	—	—

Differences between groups were estimated using the chi^2^ or Mann-Whitney *U* test, as appropriate. LTBI: latent tuberculosis infection; MDR: multidrug resistant; MTB: meningeal tuberculosis; N/A: not applicable; ND: not determined; PTB: active pulmonary tuberculosis; SD: standard deviation.

**Table 2:** Clinical and laboratory characteristics of TBM patients.

Characteristic	*N* = 10
Clinical manifestations	
Fever	5 (50)
Meningeal signs	7 (70)
Focal deficit	6 (60)
Motor	5 (50)
Sensitive	3 (30)
Cognitive decline	3 (30)
Duration of symptoms (days)	44 (31–68)
GCS on admission, median (range)	14 (7–15)
BMRC stage, median (range)	1 (0–3)
History of PTB	2 (20)
Abnormal chest X-ray	4 (40)
CSF analysis	
Leukocytes (cells/mm^3^)	256.5 (30.75–614.5)
Neutrophils (%)	4 (2.2–7.0)
Lymphocytes (%)	95.5 (51–99)
Glucose (mg/dL)	40 (24–44)
Proteins (mg/dL)	100 (66–198)
ADA (U/L)	11 (2.2–17.7)
Positive culture	2 (20)
Brain MRI	
Vasculitis	4 (40)
Hydrocephalus	2 (20)
Basal meningeal enhancement	6 (60)

Data are displayed as median (IQR) or *n* (%). ADA: adenosine deaminase; BMRC: British Medical Research Council; CSF: cerebrospinal fluid; CT: computed tomography; GCS: Glasgow Coma Scale; MRI: magnetic resonance imaging; PTB: pulmonary tuberculosis; TBM: tuberculous meningitis.

## Data Availability

The data used to support the findings of this study are available from the corresponding authors upon request.

## Supplementary Materials

Supplementary Materials Figure S1: correlation between clinical characteristics and NK cell subsets in patients with tuberculous meningitis. Figure S2: phenotype of circulating NK cells in peripheral blood mononuclear cell (PBMC) samples from patients with probable or definite tuberculous meningitis (TBM, *n* = 10).

## References

[1] World Health Organization (2019). *Global Tuberculosis Report 2019*.

[2] Cohen A., Mathiasen V. D., Schön T., Wejse C. (2019). The global prevalence of latent tuberculosis: a systematic review and meta-analysis. *European Respiratory Journal*.

[3] Loddenkemper R., Lipman M., Zumla A. (2015). Clinical aspects of adult tuberculosis. *Cold Spring Harbor Perspectives in Medicine*.

[4] Török M. E. (2015). Tuberculous meningitis: advances in diagnosis and treatment. *British Medical Bulletin*.

[5] Rock R. B., Olin M., Baker C. A., Molitor T. W., Peterson P. K. (2008). Central nervous system tuberculosis: pathogenesis and clinical aspects. *Clinical Microbiology Reviews*.

[6] Steigler P., Verrall A. J., Kirman J. R. (2019). Beyond memory T cells: mechanisms of protective immunity to tuberculosis infection. *Immunology and Cell Biology*.

[7] Kagina B. M., Abel B., Scriba T. J. (2010). Specific T cell frequency and cytokine expression profile do not correlate with protection against tuberculosis after bacillus Calmette-Guérin vaccination of newborns. *American Journal of Respiratory and Critical Care Medicine*.

[8] Choreño-Parra J. A., Weinstein L. I., Yunis E. J., Zúñiga J., Hernández-Pando R. (2020). Thinking outside the box: innate- and B cell-memory responses as novel protective mechanisms against tuberculosis. *Frontiers in Immunology*.

[9] Choreno Parra J. A., Martinez Zuniga N., Jimenez Zamudio L. A., Jimenez Alvarez L. A., Salinas Lara C., Zuniga J. (2017). Memory of natural killer cells: a new chance against Mycobacterium tuberculosis?. *Frontiers in Immunology*.

[10] Nemes E., Khader S. A., Swanson R. V., Hanekom W. A. (2020). Targeting unconventional host components for vaccination-induced protection against TB. *Frontiers in Immunology*.

[11] Khader S. A., Divangahi M., Hanekom W. (2019). Targeting innate immunity for tuberculosis vaccination. *The Journal of Clinical Investigation*.

[12] Wang X., Peng H., Tian Z. (2019). Innate lymphoid cell memory. *Cellular & Molecular Immunology*.

[13] Esin S., Batoni G. (2015). Natural killer cells: a coherent model for their functional role in Mycobacterium tuberculosis infection. *Journal of Innate Immunity*.

[14] Portevin D., Via L. E., Eum S., Young D. (2012). Natural killer cells are recruited during pulmonary tuberculosis and their ex vivo responses to mycobacteria vary between healthy human donors in association with KIR haplotype. *Cellular Microbiology*.

[15] Choreño-Parra J. A., Jiménez-Álvarez L. A., Muñoz-Torrico M. (2020). Antigens of Mycobacterium tuberculosis stimulate CXCR6+ natural killer cells. *Frontiers in Immunology*.

[16] Batoni G., Esin S., Favilli F. (2005). Human CD56^bright^ and CD56^dim^ natural killer cell subsets respond differentially to direct stimulation with Mycobacterium bovis bacillus Calmette-Guerin. *Scandinavian Journal of Immunology*.

[17] Esin S., Counoupas C., Aulicino A. (2013). Interaction of Mycobacterium tuberculosis cell wall components with the human natural killer cell receptors NKp44 and Toll-like receptor 2. *Scandinavian Journal of Immunology*.

[18] Vankayalapati R., Garg A., Porgador A. (2005). Role of NK cell-activating receptors and their ligands in the lysis of mononuclear phagocytes infected with an intracellular bacterium. *Journal of Immunology*.

[19] Feng C. G., Kaviratne M., Rothfuchs A. G. (2006). NK cell-derived IFN-gamma differentially regulates innate resistance and neutrophil response in T cell-deficient hosts infected with Mycobacterium tuberculosis. *Journal of Immunology*.

[20] Garand M., Goodier M., Owolabi O., Donkor S., Kampmann B., Sutherland J. S. (2018). Functional and phenotypic changes of natural killer cells in whole blood during Mycobacterium tuberculosis infection and disease. *Frontiers in Immunology*.

[21] Roy Chowdhury R., Vallania F., Yang Q. (2018). A multi-cohort study of the immune factors associated with M. tuberculosis infection outcomes. *Nature*.

[22] Bozzano F., Costa P., Passalacqua G. (2009). Functionally relevant decreases in activatory receptor expression on NK cells are associated with pulmonary tuberculosis in vivo and persist after successful treatment. *International Immunology*.

[23] Barcelos W., Sathler-Avelar R., Martins-Filho O. A. (2008). Natural killer cell subpopulations in putative resistant individuals and patients with active *Mycobacterium tuberculosis* infection. *Scandinavian Journal of Immunology*.

[24] Fan R., Xiang Y., Yang L. (2016). Impaired NK cells’ activity and increased numbers of CD4 + CD25+ regulatory T cells in multidrug-resistant *Mycobacterium tuberculosis* patients. *Tuberculosis*.

[25] Fu X., Yu S., Yang B., Lao S., Li B., Wu C. (2016). Memory-like antigen-specific human NK cells from TB pleural fluids produced IL-22 in response to IL-15 or *Mycobacterium tuberculosis* antigens. *PLoS One*.

[26] Fu X., Liu Y., Li L. (2011). Human natural killer cells expressing the memory-associated marker CD45RO from tuberculous pleurisy respond more strongly and rapidly than CD45RO-natural killer cells following stimulation with interleukin-12. *Immunology*.

[27] Venkatasubramanian S., Cheekatla S., Paidipally P. (2017). IL-21-dependent expansion of memory-like NK cells enhances protective immune responses against Mycobacterium tuberculosis. *Mucosal Immunology*.

[28] Esaulova E., Das S., Singh D. K. (2021). The immune landscape in tuberculosis reveals populations linked to disease and latency. *Cell Host & Microbe*.

[29] Chackerian A. A., Alt J. M., Perera T. V., Dascher C. C., Behar S. M. (2002). Dissemination of Mycobacterium tuberculosis is influenced by host factors and precedes the initiation of T-cell immunity. *Infection and Immunity*.

[30] Marais S., Thwaites G., Schoeman J. F. (2010). Tuberculous meningitis: a uniform case definition for use in clinical research. *The Lancet Infectious Diseases*.

[31] Hernandez Pando R., Aguilar D., Cohen I. (2010). Specific bacterial genotypes of Mycobacterium tuberculosis cause extensive dissemination and brain infection in an experimental model. *Tuberculosis*.

[32] Vivier E., Tomasello E., Baratin M., Walzer T., Ugolini S. (2008). Functions of natural killer cells. *Nature Immunology*.

[33] Cibrián D., Sánchez-Madrid F. (2017). CD69: from activation marker to metabolic gatekeeper. *European Journal of Immunology*.

[34] Lanier L. L. (2008). Up on the tightrope: natural killer cell activation and inhibition. *Nature Immunology*.

[35] Marcenaro E., Ferranti B., Falco M., Moretta L., Moretta A. (2008). Human NK cells directly recognize Mycobacterium bovis via TLR2 and acquire the ability to kill monocyte-derived DC. *International Immunology*.

[36] Sivori S., Falco M., Chiesa M. D. (2004). CpG and double-stranded RNA trigger human NK cells by Toll-like receptors: induction of cytokine release and cytotoxicity against tumors and dendritic cells. *Proceedings of the National Academy of Sciences of the United States of America*.

[37] Garg A., Barnes P. F., Porgador A. (2006). Vimentin expressed on Mycobacterium tuberculosis-infected human monocytes is involved in binding to the NKp46 receptor. *Journal of Immunology*.

[38] Harris L. D., Khayumbi J., Ongalo J. (2020). Distinct human NK cell phenotypes and functional responses to Mycobacterium tuberculosis in adults from TB endemic and non-endemic regions. *Frontiers in Cellular and Infection Microbiology*.

[39] Das H., Groh V., Kuijl C. (2001). MICA engagement by human V*γ*2V*δ*2 T cells enhances their antigen-dependent effector function. *Immunity*.

[40] Champsaur M., Lanier L. L. (2010). Effect of NKG2D ligand expression on host immune responses. *Immunological Reviews*.

[41] Rich A. R. (1933). The pathogenesis of tuberculous meningitis. *Bulletin of the Johns Hopkins Hospital*.

[42] Jain S. K., Paul-Satyaseela M., Lamichhane G., Kim K. S., Bishai W. R. (2006). Mycobacterium tuberculosis invasion and traversal across an in vitro human blood-brain barrier as a pathogenic mechanism for central nervous system tuberculosis. *The Journal of Infectious Diseases*.

[43] van Leeuwen L. M., Boot M., Kuijl C. (2018). Mycobacteria employ two different mechanisms to cross the blood-brain barrier. *Cellular Microbiology*.

[44] Be N. A., Lamichhane G., Grosset J. (2008). Murine model to study the invasion and survival of Mycobacterium tuberculosis in the central nervous system. *The Journal of Infectious Diseases*.

[45] Daneman R., Prat A. (2015). The blood-brain barrier. *Cold Spring Harbor Perspectives in Biology*.

[46] Kim K. S. (2008). Mechanisms of microbial traversal of the blood–brain barrier. *Nature Reviews Microbiology*.

[47] van Sorge N. M., Doran K. S. (2012). Defense at the border: the blood-brain barrier versus bacterial foreigners. *Future Microbiology*.

[48] Sánchez-Garibay C., Hernández-Campos M. E., Tena-Suck M. L., Salinas-Lara C. (2018). Experimental animal models of central nervous system tuberculosis: a historical review. *Tuberculosis*.

[49] Almerigogna F., Fassio F., Giudizi M. G. (2011). Natural killer cell deficiencies in a consecutive series of children with herpetic encephalitis. *International Journal of Immunopathology and Pharmacology*.

[50] Adler H., Beland J. L., Del-Pan N. C., Kobzik L., Sobel R. A., Rimm I. J. (1999). In the absence of T cells, natural killer cells protect from mortality due to HSV-1 encephalitis. *Journal of Neuroimmunology*.

[51] Shieh T. M., Carter D. L., Blosser R. L., Mankowski J. L., Zink M. C., Clements J. E. (2001). Functional analyses of natural killer cells in macaques infected with neurovirulent simian immunodeficiency virus. *Journal of Neurovirology*.

[52] Stach J. L., Dufrenoy E., Roffi J., Bach M. A. (1986). T-cell subsets and natural killer activity in Plasmodium falciparum-infected children. *Clinical Immunology and Immunopathology*.

[53] Hayashi T., Nagai S., Fujii H. (2009). Critical roles of NK and CD8+ T cells in central nervous system listeriosis. *Journal of Immunology*.

[54] Mitchell A. J., Yau B., McQuillan J. A. (2012). Inflammasome-dependent IFN-*γ* drives pathogenesis in Streptococcus pneumoniae meningitis. *Journal of Immunology*.

[55] Rao D., Venkataswamy M. M., Vasanthapuram R., Satishchandra P., Desai A. (2018). Alterations in natural killer and dendritic cell subsets in individuals with HIV-associated neurotuberculosis. *Journal of Medical Virology*.

[56] van Laarhoven A., Dian S., van Dorp S. (2019). Immune cell characteristics and cytokine responses in adult HIV-negative tuberculous meningitis: an observational cohort study. *Scientific Reports*.

