# Supplementary material for: Phenotype of Peripheral NK Cells in Latent, Active, and Meningeal Tuberculosis
Source: J Immunol Res. 2021 Apr 27;2021:5517856. doi: 10.1155/2021/5517856 (PMC8100419; doi:10.1155/2021/5517856)
Supplement: Supplementary Materials — Figure S1: correlation between clinical characteristics and NK cell subsets in patients with tuberculous meningitis. Figure S2: phenotype of circulating NK cells in peripheral blood mononuclear cell (PBMC) samples from patients with probable or definite tuberculous meningitis (TBM, n = 10). [file 5517856.f1.docx]

**Supplementary Material**

**
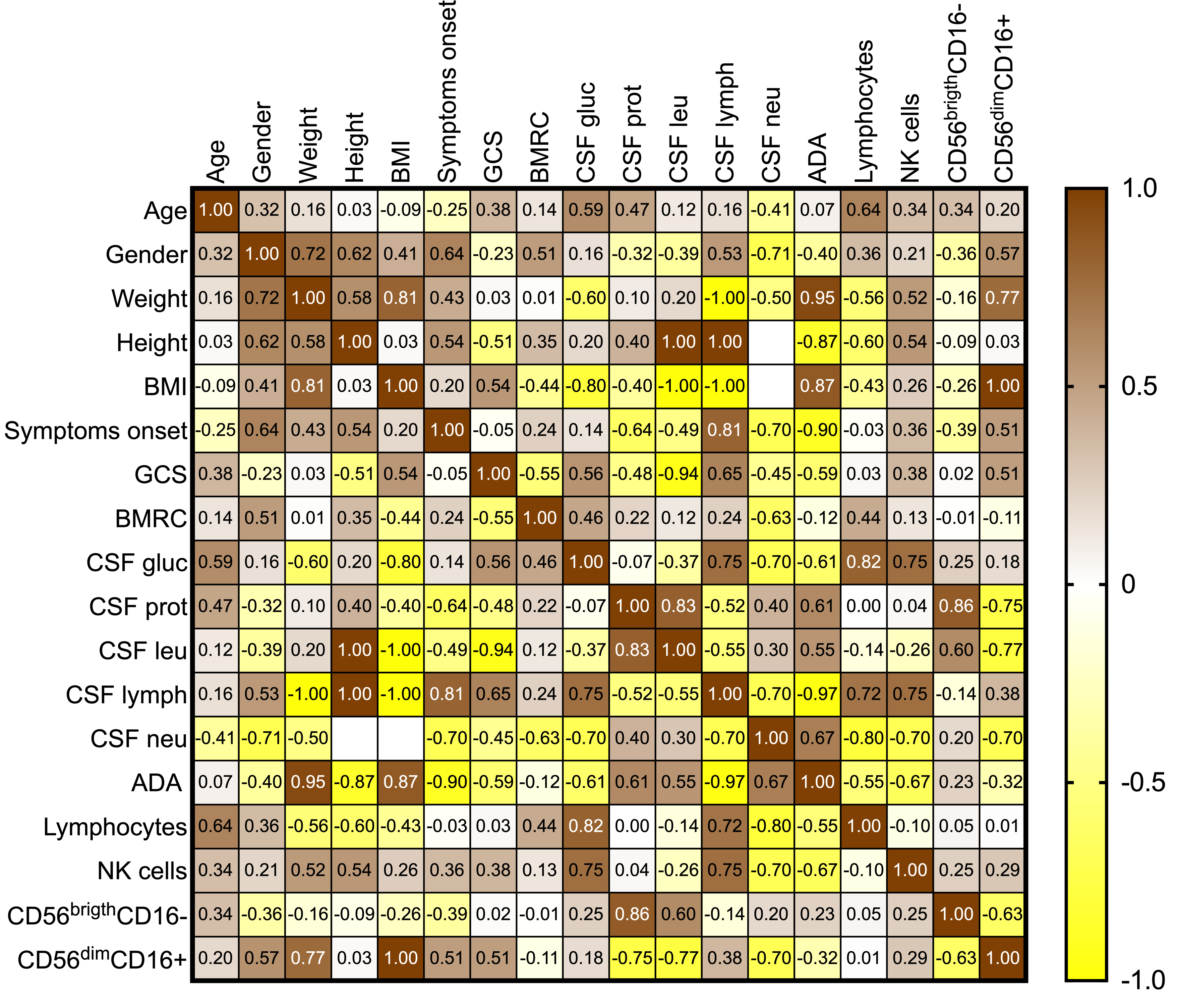
**

**Figure S1. Correlation between clinical characteristics and NK cell subsets in patients with tuberculous meningitis.** The heat-map displays Spearman correlation coefficients between clinical variables and NK cells. ADA, adenosine deaminase; BMI, body mass index; BMRC, British Medical Research Council stage; CSF, cerebrospinal fluid; GCS, Glasgow Coma Scale.

**
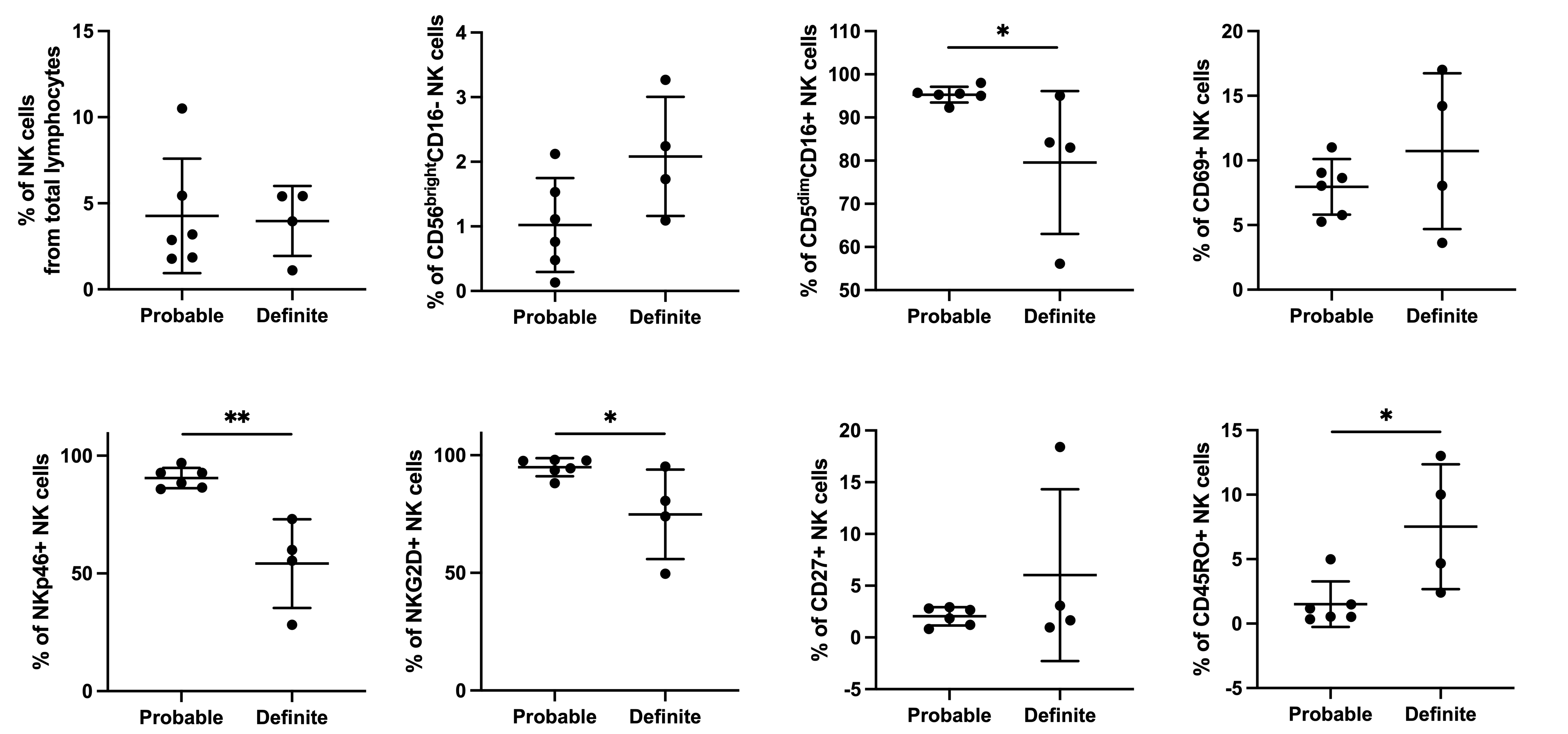
**

**Figure S2.** Phenotype of circulating NK cells in peripheral blood mononuclear cell (PBMCs) samples from patients with probable or definite tuberculous meningitis (TBM, n = 10). Differences between groups were analyzed using the Mann-Whitney U test and Student T test, as appropriate. The data shown represent mean (±SE) values. *p≤0.05, **p≤0.01.
